# Assessment of oral health in older adults by non-dental professional caregivers—development and validation of a photograph-supported oral health–related section for the interRAI suite of instruments

**DOI:** 10.1007/s00784-020-03669-8

**Published:** 2020-11-16

**Authors:** Stefanie Krausch-Hofmann, Trung Dung Tran, Barbara Janssens, Dominique Declerck, Emmanuel Lesaffre, Johanna de Almeida Mello, Anja Declercq, Jan De Lepeleire, Joke Duyck

**Affiliations:** 1 KU Leuven Department of Oral Health Sciences, Population Studies in Oral Health, Kapucijnenvoer 7/a - box 7001, 3000 Leuven, Belgium; 2 KU Leuven Department of Public Health and Primary Care, Biostatistics and Statistical Bioinformatics Centre (L-BioStat), Kapucijnenvoer 35/a - box 7001, 3000 Leuven, Belgium; 3 Ghent University Department of Oral Health Sciences, Special Needs in Oral Health, Gerodontology, C.-Heymanslaan 10, entrance 25, 9000 Ghent, Belgium; 4 KU Leuven LUCAS, Centre for Care Research and Consultancy, Minderbroedersstraat 8 - box 5310, 3000 Leuven, Belgium; 5 KU Leuven CESO, Center for Sociological Research, Parkstraat 45 - box 3601, 3000 Leuven, Belgium; 6 KU Leuven Department of Public Health and Primary Care, Academic Centre for General Practice, Kapucijnenvoer 33/j - box 7001, 3000 Leuven, Belgium; 7 KU Leuven Department of Oral Health Sciences, Biomaterials/BIOMAT, Kapucijnenvoer 7/a - box 7001, 3000 Leuven, Belgium

**Keywords:** Oral health assessment, Oral screening, Older care-dependent adults, Non-dental caregivers, InterRAI suite of instruments

## Abstract

**Objectives:**

An optimized oral health-related section and a video training were developed and validated for the interRAI suite of instruments. The latter is completed by professional non-dental caregivers and used in more than 40 countries to assess care needs of older adults.

**Methods:**

The optimized oral health–related section (ohr-interRAI) consists of nine items and a video training that were developed in consecutive phases. To evaluate psychometric properties, a study was conducted in 260 long-term care residents. Each resident was assessed by a dentist and by four caregivers (two who received the video training, two who did not).

**Results:**

Mean kappa values and percent agreement between caregivers and dentist ranged between *κ* = 0.60 (80.2%) for *dry mouth* and *κ* = 0.13 (54.0%) for *oral hygiene*. The highest inter-caregiver agreement was found for *dry mouth* with *κ* = 0.63 [95% CI: 0.56–0.70] (81.6%), while for the item *palate/lips/cheeks* only *κ* = 0.27 [95% CI: 0.18–0.36] (76.7%) was achieved. Intra-caregiver agreement ranged between *κ* = 0.93 [95% CI: 0.79–1.00] (96.4%) for *dry mouth* and *κ* = 0.45 [95% CI: 0.06-0.84] (82.8%) for *gums.* Logistic regression analysis showed only small differences between caregivers who watched the video training and those who did not.

**Conclusions:**

Psychometric properties of the optimized ohr-interRAI section were improved compared to previous versions. Nevertheless, particularly the items based on inspection of the mouth require further refinement and caregiver training needs to be improved.

**Clinical Relevance:**

Valid assessment of oral health by professional caregivers is essential due to the impaired accessibility of regular dental care for care-dependent older adults.

**Supplementary Information:**

The online version contains supplementary material available at 10.1007/s00784-020-03669-8.

## Introduction

International research consistently reports poor oral health in care-dependent older individuals [1–4]. This causes oral infection and tooth loss and finally results in compromised oral functioning [5] and reduced quality of life [6, 7]. Oral health is further associated with the general cognitive and physical condition [8–10], and with systemic diseases such as diabetes mellitus or cardiovascular disease [11–13]. To maintain good oral health, daily oral care and regular professional check-ups and cleanings are essential. In care-dependent older adults, realization of these measures is challenged by cognitive and physical impairment and by accessibility and availability of care [14].

Non-dental professional caregivers can play a role in oral health screening to facilitate improvement of daily oral hygiene and timely referral to dental care [15, 16]. A variety of screening instruments is available such as the Oral Health Assessment Tool (OHAT) [16], the Revised Oral Assessment Guide (ROAG) [17], the Oral Health Screening Tool for Nursing Personnel (OHSTNP) [18], or the oral health-related section of the Minimum Data Set 2.0/interRAI suite of instruments (ohr-MDS 2.0/ohr-interRAI) [19]. Studies on psychometric properties of the above instruments vary with regard to the professional background of the caregivers, how they were trained, or to what benchmark their registrations were compared [16, 17, 20–24]. This variability and methodological shortcomings of the studies impair a reliable comparison between the oral screening tools [25].

A practical challenge for the effectiveness of any oral screening tool is its broad implementation in everyday care. Only if oral health of all clients is assessed regularly, care needs can be detected and tackled. While most screening instruments were developed and applied within a rather academic context, the ohr-interRAI and related precursor versions are widely used in more than 30 countries in North America, Europe, Asia, and the Pacific Rim [26]. The ohr-interRAI section belongs to the interRAI suite of instruments that consists of tools for comprehensive assessment of care needs. Various aspects of health and well-being are evaluated and used for holistic care planning. It is completed by caregivers upon admission to residential care or home care services and repeated periodically. A valid oral health section holds the potential to integrate oral care into general care planning. The current interRAI version for long-term care consists of six dichotomous (yes/no) oral health items that register removable dental prosthesis use, non-intact teeth, dry mouth, chewing problems, gum inflammation and pain. Caregivers can choose to examine, interview, or observe clients, but clear definitions or guidelines on how to assess oral health are not provided in the utilization manual [19]. Compared to its precursor version included in the Minimum Data Set 2.0, the ohr-interRAI was slightly shortened and modified. [The different versions are shown in the supplementary material of this article].

Although widely used, it was consistently shown that the current ohr-interRAI section and related precursor versions do not adequately detect oral health-related care needs [24, 27–32]. In a study on the underlying reasons for this failure, experts challenged completeness, relevance, clarity of wording, and feasibility of the items [33]. Focus group discussions with caregivers revealed further shortcomings of the ohr-interRAI section, situational factors that impeded the assessment, and low awareness for oral health in the care environment. It was also found that the approach of the caregivers to complete the ohr-interRAI section was not suited to accurately detect care needs [33].

The present study aimed to develop and validate an optimized, photograph-supported ohr-interRAI section that effectively detects clients who need assistance with daily oral hygiene and/or referral to a dentist.

## Materials and methods

### Phase I: Development of an optimized ohr-interRAI section and a video training

Step 1: After literature review, test content, and requirements of an optimized ohr-interRAI section were discussed by a group of 12 experts. Participants had an academic-clinical background in gerodontology (*n* = 5), prosthetic dentistry (*n* = 2), periodontology (*n* = 1), geriatric medicine (*n* = 3), and geriatric nursing care (*n* = 1). There was a consensus that, as an integrated part of the comprehensive interRAI assessment, the number of items should not exceed 10. An optimized ohr-interRAI section should not only include client self-reports (chewing problems, pain/discomfort, dry mouth) but also mandate an inspection of the mouth (oral/denture hygiene and condition of teeth, gums, tongue, palate, lips, and cheeks). It was decided that the optimized ohr-interRAI section should differentiate between acceptable and non-acceptable conditions to indicate the need of a care intervention. The terminology acceptable/non-acceptable was chosen as the assessed persons are in the last phase of their life. It was considered that a meaningful and realistic oral health assessment should allow for the fact that in this population, aberrations from perfect oral health are tolerable. As a minimum standard, oral health should be acceptable. There was also a consensus that exemplary photographs—clearly and consistently interpreted by oral health professionals—were needed as visualizations for oral screening.
Step 2: Preliminary items were formulated. To define acceptable and non-acceptable conditions for each item, literature was reviewed and several discussion rounds among the members of the research group were organized. Related to *chewing problems*, *pain/discomfort*, and *dry mouth*, definition of acceptable and non-acceptable conditions proved difficult. For these items, the response category non-acceptable was preliminarily divided into moderate and marked, anticipating that a refined dichotomous version will be formulated later based on the results and the experience of the presented research. Moreover, general utilization guidelines, definitions, and item-wise instructions on how to do the oral health assessment were added.
Step 3: Photographs to visualize the 6 items that require inspection of the mouth were taken from older long-term care residents and from patients at the Department of Oral Health Sciences, University Hospitals Leuven. For each item, about 30 photographs were collected, including a variety of conditions that ranged from healthy to severely unhealthy. The photographs further depicted different views, such as the dorsal, lateral, and ventral surface of the tongue. The condition shown on each of the 179 photographs was then assessed by oral health professionals to exclude the most unclear visualizations. Three university-associated dentists, specialized in gerodontology, special needs dentistry, and periodontology, provided a collective score that constituted the benchmark. Photographs were further individually assessed by 32 general dentists. Only those photographs were considered to be used as visualizations if at least 80% of the dentists agreed with the benchmark on whether acceptable or non-acceptable conditions were shown. According to Hugh (2012), 80% is widely accepted and recommended in the literature as minimum standard for agreement among raters [34].
Step 4: In-depth interviews with 7 non-dental caregivers—acquainted with the current ohr-interRAI section—were conducted to refine the preliminary photograph-supported items and guidelines. (The items and utilization guidelines of the optimized ohr-interRAI section are shown in the supplementary material of this article.)
Step 5: A video training was produced that consisted of 9 clips with a total duration of about 30 minutes. It included comprehensive information on the oral health assessment and a variety of photographs. The first video illustrated the relevance of oral health and introduced known associations between oral health and general health. In the second video, general utilization guidelines of the optimized ohr-interRAI section were clarified and video 3 was about the registration of *chewing problems*, *pain/discomfort* and *dry mouth*. The remaining videos covered each an individual item: 4 *denture hygiene*, 5 *oral hygiene*, 6 *teeth*, 7 *gums*, 8 *tongue*, and 9 *palate/lips/cheeks*. (The videos are available in the supplementary material of this article.)

### Phase II: Study to evaluate psychometric properties of the optimized ohr-interRAI

Management executives of all 137 long-term care facilities in the province of Flemish-Brabant, Belgium, were invited by e-mail to participate. From each of the 9 facilities that agreed to take part, 4 non-dental caregivers were recruited. Before commencing the study, caregivers completed a questionnaire with 30 statements to evaluate oral health-related knowledge. The statements included oral health–general health associations and the appearance of oral health diseases. During a 1-hour session, all caregivers received instructions on completion of the optimized ohr-interRAI section and the study procedure. Two caregivers from each facility watched the video training in addition. After finishing data collection which took 2 or 3 days per care facility, caregivers again completed the questionnaire on oral health-related knowledge. Two dentist researchers examined the residents. To calibrate dentists, 20 residents were examined twice and differences were discussed per item. For the first 10 of these residents, a third dentist was present for calibration.

A convenience sample of non-palliative residents was taken. Sample size calculation was based on a binomial distribution of item responses, a worst-case theoretical detection ability of 50% and an acceptable estimation error of about 7%. It was determined that at least 200 residents had to be included. An extra 30% of residents were added to account for potential dropouts during data collection.

For each resident, all oral health registrations were performed on the same day, spread over a period of about 3 hours. It was estimated that the total duration of the assessments summed up to approximately 35 minutes, depending on her or his dental status. Residents were individually assessed in their private room by each of the 4 caregivers using flashlights for illumination. The sequence of residents was scheduled for each caregiver to prevent order bias and to ensure efficiency of the data collection procedure. In addition, about 10% of the residents were assessed a second time by one of the caregivers. Finally, residents were examined by a dentist. First, the dentist inspected the mouth visually without additional aids, only using a headlamp for illumination. This corresponded to the conditions that applied to the caregivers. In a second step, dentists repeated the assessment using a standard dental mirror and a dental explorer. These registrations were considered the benchmark. After the oral health registration, the dentist rated the assessment ease to provide an estimate of how well the participant could be assessed. Considering communication fluency and physical cooperation, the dentist indicated a single value on a 5-point Likert scale that ranged from very easy to very difficult.

### Statistical analysis

Items with three response categories were dichotomized as follows: 1 = acceptable, 2 = non-acceptable moderate, and 3 = non-acceptable marked. Psychometric properties were determined for the individual items. They were further quantified for the resulting interventions *assistance with hygiene* and *referral to dentist* that were established by the individual items as follows:

- *Assistance with hygiene*: *denture hygiene*, *oral hygiene*
- *Referral to dentist*: *chewing problems*, *pain/discomfort*, *dry mouth*, *teeth*, *gums*, *tongue*, *palate/lips/cheeks*.

Interventions were recommended if one or more of the constituting items were rated as non-acceptable.

Agreement of caregivers with the dentist (using dental mirror and explorer), inter-caregiver, and intra-caregiver were quantified using percent agreement and kappa statistics (Cohen’s kappa and Krippendorff’s alpha). For kappa statistics, the following interpretation was used: 0–0.20 no, 0.21–0.39 minimal, 0.40–0.59 weak, 0.60–0.79 moderate, 0.80–0.90 strong, and >0.90 almost perfect agreement [34]. Observations were excluded from the analysis if assessment of the condition was not possible due to non-cooperation of the resident or if dentures, retainers, or teeth were not present (outcome absent).

Logistic regression was used to model the effect of the video training on dentist-caregiver agreement and on inter-caregiver agreement. Fixed and random effects were included for residents and care facilities. Models were corrected for the following covariates: gender, age, and assessment ease of residents as well as gender, age, previous attendance of continuing education activities on oral health, responsibility for daily oral care, and work experience of caregivers.

Statistical programs SPSS (version 23), SAS (version 9.4), and R (version 3.6) were used.

## Results

### Descriptive results

The optimized ohr-interRAI section that was developed in the context of this study consists of 9 photograph-supported items, utilization guidelines, and a video training. Originally developed and tested in the Flemish-Dutch language, the instrument was translated to English for the readers of this journal (see supplementary material of this article).

The study that evaluated the psychometric properties of the developed instrument was conducted in 9 long-term care facilities in Flanders, Belgium. They had a capacity between 43 and 131 beds; 5 were public and 4 were privately organized.

Thirty-six caregivers—4 from each care facility—participated in the study. A proportion of 83.3% were female and they belonged to the following age groups: <30 years = 27.8%, 30–40 years = 38.9%, 41–50 years = 8.3%, and >50 years = 25.0%. With regard to professional function, 52.8% were nurses, 25.0% nurse aids, 16.7% occupational therapists, and 5.6% speech therapists. Previous continuous education activities on oral health were attended by 25.0% and 75.0% provided daily oral care for clients.

Between 24 and 30 residents from each care facility participated in the study—260 in total. The mean age was 86.3 (± 7.3) years and 76.9% was female. While 57.4% could be assessed very easy or easy, 28.3% were classified as neutral and 14.3% were difficult or very difficult to examine.

Tables 1 and 2 present oral health and hygiene and the resulting interventions for dentists and caregivers, respectively. For the shown registrations, dentists had used a dental mirror and an explorer. Without these aids, about 6% less non-acceptable conditions were detected by dentists for *oral hygiene*, *teeth*, and *gums*. For *tongue* and *palate/lips/cheeks*, the difference was about 1%.

**Table 1:** Oral health and hygiene of residents registered by dentists and caregivers using the optimized ohr-interRAI section

	Dentist						Caregivers^2^					
	Acceptable		Non-acceptable		Outcome absent^3^		Acceptable		Non-acceptable		Outcome absent^3^	
	*n*	%	*n*	%	*n*	%	*n*	%	*n*	%	*n*	%
Chewing problems*	103	39.6	145	55.8	12	4.6	108	42.0	138	53.4	12	4.6
Pain/discomfort*	214	82.3	29	11.2	17	6.5	213	82.9	37	14.3	7	2.7
Dry mouth*	136	52.3	106	40.8	18	6.9	126	48.9	123	47.9	9	3.2
Denture hygiene+	80	30.8	100	38.5	80	30.8	136	53.8	42	16.4	76	29.9
Oral hygiene+	24	9.2	130	50.0	106	40.8	90	35.6	63	24.9	100	39.5
Teeth+	35	13.5	117	45.0	108	41.5	57	22.2	97	37.6	103	40.3
Gums+^1^	116	44.6	137	52.7	7	2.7	197	76.9	56	21.6	4	1.5
Tongue+	221	85.0	26	10.0	13	5.0	195	76.0	59	22.8	3	1.2
Palate/lips/cheeks+	193	74.2	59	22.7	8	3.1	205	79.8	49	18.8	8	1.4

^1^In patients without teeth or implant-based retainers, gum tissue of the edentulous ridges was assessed.

^2^Arithmetic mean over all caregivers

^3^Assessment was not possible due to the condition of the resident or dentures/retainers/teeth were not present

*Registered based on client self-reports. Assessors turned to family/other caregivers when clients were not able to communicate

^*+*^Registered based on inspection of the mouth. To collect the presented data, dentists used a standard dental mirror and a dental explorer. Caregivers did not apply any diagnostic instruments

**Table 2:** Recommended interventions based on the optimized ohr-interRAI

	Dentist				Caregivers^1^			
	No		Yes^2^		No		Yes^2^	
	*n*	%	*n*	%	*n*	%	*n*	%
Assistance with hygiene	72	27.7	188	72.3	160	64.0	91	36.0
Referral to dentist	22	8.5	238	91.5	31	12.0	226	88.0

^1^Arithmetic mean over all caregivers

^2^If ≥ 1 of the constituting items was non-acceptable

As only isolated and small effects of the video-training were found (Table 5), arithmetic means over all caregivers are reported in Tables 1, 2, 3, and 4.

**Table 3:** Dentist-caregiver agreement

	Dentist-caregiver agreement^2,3^						
	Overall			Non-acceptable/intervention recommended^4^		Acceptable/no intervention recommended^5^	
	*n*	%	*κ*	*n*	%	*n*	%
Chewing problems*	189	78.6	0.56	111	79.2	79	77.6
Pain/discomfort*	206	86.6	0.41	16	53.6	191	91.1
Dry mouth*	190	80.2	0.60	89	84.7	102	76.7
Denture hygiene+	106	61.8	0.28	35	36.7	71	92.9
Oral hygiene+	71	54.0	0.13	55	49.7	16	77.1
Teeth+	102	69.9	0.29	82	72.2	21	62.1
Gums+^1^	138	55.8	0.15	40	29.4	99	86.7
Tongue+	187	76.5	0.18	13	47.6	174	79.9
Palate/lips/cheeks+	184	74.0	0.23	21	35.3	164	85.9
Assistance with hygiene	141	56.1	0.23	81	44.8	60	86.7
Referral to dentist	228	87.7	0.39	216	92.0	12	54.5

^1^In patients without teeth or implant-based retainers, gum tissue of the edentulous ridges was assessed.

^2^Arithmetic mean over all caregivers

^3^Registrations were not considered in the analysis if the outcome was absent.

^4^Calculation according to sensitivity

^5^Calculation according to specificity

*Registered based on client self-reports. Assessors turned to family/other caregivers when clients were not able to communicate

^+^Registered based on inspection of the mouth. To collect the presented data, dentists used a standard dental mirror and a dental explorer. Caregivers did not apply any diagnostic instruments

**Table 4:** Inter-caregiver and intra-caregiver agreement

	Inter-caregiver agreement^2^		Intra-caregiver agreement^2^	
	%^3^	*κ* [95% CI]	%	*κ* [95% CI]
Chewing problems	76.6	0.53 [0.45–0.60]	96.4	0.92 [0.78–1.00]
Pain/discomfort	87.8	0.51 [0.39–0.61]	85.7	0.63 [0.32–0.94]
Dry mouth	81.6	0.63 [0.56–0.70]	96.4	0.93 [0.79–1.00]
Denture hygiene	77.3	0.38 [0.27–0.48]	90.0	0.62 [0.15–1.00]
Oral hygiene	69.8	0.36 [0.26–0.46]	81.3	0.63 [0.24–1.00]
Teeth	74.4	0.45 [0.35–0.55]	80.0	0.60 [0.20–1.00]
Gums^1^	74.7	0.28 [0.19–0.36]	82.8	0.45 [0.06–0.84]
Tongue	73.2	0.27 [0.19–0.35]	86.2	0.66 [0.35–0.96]
Palate/lips/cheeks	76.7	0.27 [0.18–0.36]	96.4	0.90 [0.71–1.00]
Assistance with hygiene	72.1	0.39 [0.32–0.46]	82.8	0.59 [0.26–0.91]
Referral to dentist	87.6	0.42 [0.29–0.52]	93.1	0.63 [0.15–1.00]

^1^In patients without teeth or implant-based retainers, gum tissue of the edentulous ridges was assessed

^2^Registrations were not considered in the analysis if the outcome was absent

^3^Arithmetic mean over all caregivers

### Dentist-caregiver agreement

Over all caregivers, overall agreement with the benchmark was the highest for those items that were based on client self-reports (Table 3). Kappa values (*κ*) and percent agreement are provided to quantify agreement. For *dry mouth*, *chewing problems*, and *pain/discomfort*, the mean dentist-caregiver agreement was *κ* = 0.60 (80.2%), *κ* = 0.56 (78.6%), and *κ* = 0.41 (86.6%), respectively. For the remaining items, mean agreement ranged between *κ* = 0.29 (69.9%) for *teeth* and *κ* = 0.13 (54.0%) for *oral hygiene*. With regard to the resulting interventions, mean agreement was *κ* = 0.39 (87.7%) for *referral to dentist* and *κ* = 0.23 (56.1%) for *assistance with hygiene*.

### Inter- and intra-caregiver agreement

As shown in Table 4, the highest inter- and intra-caregiver agreement was reached for the items based on client self-reports.

*Dry mouth* scored best with *κ* = 0.63 [95% CI 0.56–0.70] (81.6%), indicating a moderate inter-caregiver agreement, whereas for the item on *palate/lips/cheeks* only *κ* = 0.27 [95% CI 0.18–0.36] (76.7%) was found.

Intra-caregiver agreement was highest for *dry mouth* with *κ* = 0.93 [95% CI 0.79–1.00] (96.4%), and lowest for *gums* with *κ* = 0.45 [95% CI 0.06–0.84] (82.8%)*.*

### Effect of the video training

With regard to caregivers’ oral health-related knowledge, the mean increase of the number of correct answers was 4.17 (± 3.37) for those without the video training, and 10.44 (± 6.43) for those who had watched the videos.

Table 5 illustrates agreement differences between both groups of caregivers. If caregivers had watched the video training, agreement with the dentist was significantly higher for non-acceptable *oral hygiene* (OR = 1.95 [95% CI 1.16–3.27]) and *gums* (OR = 1.69 [95% CI 1.01–2.85]). However, lower agreement with the dentist was found for acceptable *gums* (OR = 0.47 [95% CI 0.23–0.95]) and *tongue* (OR = 0.65 [95% CI 0.76–1.19]).

**Table 5:** Differences between caregivers with/without video training

	Differences between caregivers with/without video training					
	Dentist-caregiver agreement				Inter-caregiver agreement	
	Non-acceptable/intervention recommended		Acceptable/no intervention recommended			
Item/combination	Odds ratio [95% CI]	*P* value	Odds ratio [95% CI]	P-value	Odds ratio [95% CI]	*P* value
Chewing problems	0.89 [0.49–1.62]	0.708	1.86 [0.97–3.55]	0.062	1.52 [0.98–2.37]	0.065
Pain/discomfort	0.90 [0.24–3.36]	0.873	1.69 [0.87–3.30]	0.125	0.98 [0.57–1.66]	0.927
Dry mouth	0.95 [0.49–1.84]	0.869	1.83 [0.97–3.47]	0.064	1.07 [0.67–1.70]	0.775
Denture hygiene	0.93 [0.51–1.69]	0.815	1.80 [0.60–5.40]	0.295	0.65 [0.41–1.02]	0.065
Oral hygiene	1.95 [1.16–3.27]	0.012	0.56 [0.10–3.18]	0.514	1.06 [0.71–1.56]	0.788
Teeth	0.60 [0.33–1.09]	0.095	0.91 [0.37–2.29]	0.857	0.96 [0.60–1.54]	0.862
Gums^1^	1.69 [1.01–2.85]	0.047	0.47 [0.23–0.95]	0.037	0.91 [0.60–1.38]	0.672
Tongue	0.63 [0.23–1.77]	0.388	0.65 [0.76–1.19]	0.035	1.54 [1.01–2.34]	0.045
Palate/lips/cheeks	1.40 [0.65–3.01]	0.385	0.57 [0.32–1.03]	0.063	0.80 [0.51–1.23]	0.302
Assistance with hygiene	1.49 [0.92–2.12]	0.112	0.93 [0.37–2.32]	0.869	1.22 [0.78–1.89]	0.381
Referral to dentist	0.94 [0.53–1.69]	0.840	1.88 [0.46–7.59]	0.382	1.10 [0.63–1.92]	0.738

^1^In patients without teeth or implant-based retainers, gum tissue of the edentulous ridges was assessed

Inter-caregiver agreement was significantly higher for *tongue* (OR = 1.54 [95% CI 1.01–2.34]) for caregivers with video training.

## Discussion

An optimized oral health–related section was developed for the interRAI suite of instruments. It consists of 9 items that include client self-reports but also require inspection of the mouth. As chewing problems, pain, and dry mouth impair oral health–related quality of life [35], registration of these aspects is crucial. However, an inspection of the mouth is inevitable since older individuals are often not aware or do not complain about oral problems [36, 37]. Photographs were used as visualizations to help caregivers to recognize non-acceptable oral conditions and to raise the awareness for oral health.

### Prevalence of oral health–related problems

Residents who participated in this study were not selected randomly, results do not refer to palliative residents, and over-representation of cooperative individuals is highly probable. However, the prevalence of problems identified by dentists confirms the findings of earlier research reporting poor oral health in care-dependent older adults in Belgium [38, 39]. In the present study, hygiene was non-acceptable for 55% of the dentures. Among residents with teeth or implant-based retainers in the mouth, 84% had non-acceptable oral hygiene. Accordingly, De Visschere et al. (2016) reported for the oldest age group that in 43% of the dentures, at least 25% of the surface was covered with plaque. The mean dental plaque score also demonstrated poor oral hygiene [38]. Seventy-seven percent of the dentate residents had a non-acceptable tooth condition in the present study, which is in line with the dental treatment needs reported by De Visschere et al. (2016) and Janssens et al. (2017) [38, 39]. In our study, a non-acceptable *gum* condition was registered in 54.1% of the residents whose gums could be assessed. De Visschere et al. (2016) found periodontal disease in 87% [38]. This considerable difference is likely to arise from the different methodologies that were applied. In contrast to De Visschere et al., in the present study, periodontal tissues were not manipulated and the mucosa on the alveolar bone was assessed in edentulous residents. With regard to mucosal tissues, non-acceptable conditions for *tongue* and *palate/lips/cheeks* were registered in 10.5% and 23.4% of the residents, respectively. Accordingly, De Visschere et al. (2016) observed mucosal lesions in about 25% of their participants [38].

Subjective oral health in care-dependent older individuals has not been evaluated in recent studies in Belgium. The present research identified 55.8% of residents with *chewing problems*. This proportion is higher compared to international studies reporting prevalence rates between 25.6 and 48.7% [40, 41]. Participating dentists in the current study remarked after data collection that *chewing problems* might be defined too strictly and suggested revision of this item. *Pain or discomfort* was registered in 11.2% of the residents which is comparable to Delwel et al. (2018) reporting oro-facial pain in 0–10% [37]. In our study, dentists registered a *dry mouth* sensation in 40.8% of the residents which is in line with other studies reporting prevalence rates between 28.6 and 63% [40, 42–45].

With regard to consistency within the current study, it might have been beneficial to have all participants examined by the same dentist. However, the calibration process revealed differences among dentists that could be mitigated by discussion. Hence, it can be assumed that using more than one dentist enhanced reproducibility of the study results.

### Dentist-caregiver agreement

Dentists and caregivers used the optimized ohr-interRAI section to assess oral health. The agreement of their registrations provides information on concurrent validity of the items. Dentists had specific experience with frail older adults, were calibrated, and used dental mirrors and explorers for the oral examination. The prevalence of oral health problems was comparable to other studies with older adults in Belgium [38, 39], confirming that dentist registrations were a valuable benchmark to compare caregiver assessments with.

In accordance with comparable research, kappa values are reported to quantify agreement after the correction for chance agreement. As kappa values cannot be interpreted straightforwardly and as they are affected by the prevalence of the registered conditions [46], percent agreement is also reported.

In the current study, the highest kappa values were found for *chewing problems* (*κ* = 0.56; 78.6%) and *dry mouth* (*κ* = 0.60; 80.2%), indicating slightly moderate dentist-caregiver agreement. Among residents with a non-acceptable *dry mouth*, 84.7% were correctly identified by caregivers, which was the case for 79.2% of the residents who had c*hewing problems*.

Agreement with the dentist on *pain/discomfort* was weak with *κ* = 0.41 (86.6%) in the current study. However, this finding represents an improvement compared to previous versions of the oral health section. Hoben et al. (2019) reported *κ* = 0.13 for the agreement between dental hygienists and trained research assistants using the MDS 2.0. Untrained care staff did not identify any residents with oral pain [47]. Under-detection of oral pain in MDS data was also confirmed by Folse (2001) [27].

For *teeth*, the current study found minimal agreement with *κ* = 0.29 (69.9%) and 72.2% correct identification of non-acceptable conditions. Hoben et al. (2019) reported *κ* = 0.49 for the agreement between dental hygienists and trained research assistants with 69.6% correct identification of tooth problems. For untrained regular care staff, *κ* = 0.02 and 4.4% correct identification was found [47]. However, direct comparison with our results is impeded as Hoben et al. (2019) included an *outcome absent* category in their analysis. Folse (2001) also found serious under-detection of dental problems when MDS data were compared to dental examination forms [27].

The present study found very low dentist-caregiver agreement for *tongue* and for *palate/lips/cheeks*. Comparison with other studies is not possible, as previous versions of the oral health–related section did not assess these aspects.

The second-lowest kappa in this study was found for *gums* (*κ* = 0.15; 55.8%). Dentists detected 137 residents with a non-acceptable condition, but only 29.4% were identified correctly by caregivers. Hoben et al. (2019) reported comparable results for the MDS 2.0 with trained research assistants who correctly identified 24.6% of residents with gum problems (*κ* = 0.14). Untrained care staff in the same study did not identify any residents with gum problems [47].

In the current study, the lowest kappa was found for *oral hygiene* (*κ* = 0.13; 54.0%). From the 130 residents with a non-acceptable condition, only 49.7% were correctly identified by the caregivers. However, dentist-caregiver agreement on oral hygiene was improved compared to previous versions. Hoben et al. (2019) reported *κ* = 0.05 for trained research assistants and *κ* = - 0.02 for nursing home staff [47]. Nordenram and Ljunggren (2002) found that from the 179 residents with non-acceptable oral hygiene detected by a dentist, only 12% was correctly identified by the MDS 2.0 [28].

A previous study on the shortcomings of the current ohr-interRAI section revealed that caregivers demanded an outcome that is derived from the oral health assessment to improve care [33]. To meet this request, two interventions were linked to the optimized ohr-interRAI section. If *denture* and/or *oral hygiene* was rated as non-acceptable, *assistance with hygiene* was recommended. *Referral to the dentist* was advised if one or more of the other items was considered non-acceptable. For both interventions, the current study found a low dentist-caregiver agreement with *κ* = 0.23 (56.1%) for *assistance with hygiene* and *κ* = 0.39 (87.7%) for *referral to dentist*. However, of the residents who were recommended to be referred to the dentist, 92.0% were correctly identified by caregivers. In contrast, Nordenram and Ljunggren (2002) reported that only 50% of residents with treatment need were correctly identified by caregivers [28].

### Inter-caregiver agreement

Kappa values in the current study ranged between *κ* = 0.27 (73.2%) and *κ* = 0.63 (81.6%), indicating minimal to slightly moderate agreement between caregivers. The highest kappas were found for the items based on subjective reports, with *κ* = 0.51 (87.8%) for *pain/discomfort* and *κ* = 0.63 for *dry mouth* (81.6%). Kappa values reported by Hoben et al. (2019) were lower with *κ* = 0.29 for chewing and *κ* = 0.20 for mouth pain [47].

For the items on *hygiene* and condition of the *teeth*, the current study found minimal to weak agreement (*κ* = 0.36–0.45; 69.8–74.4%). Hoben et al. (2019) reported a negative kappa for tooth problems and *κ* = 0.780 for debris in the mouth [47]. However, a direct comparison of results is difficult as the authors included agreement on the absence of an outcome in their analysis.

The lowest kappas were found for *gums* and the soft tissue–related items, which is in line with Hoben et al. (2019) who found *κ* = 0.27 for gum problems for trained research assistants [47].

With regard to the recommended interventions, inter-caregiver agreement was weak in the current study. Morris et al. (1997) reported moderate agreement for the MDS 2.0 with an average kappa of *κ* = 0.7 for oral-dental items [48]. However, detailed information on data collection and prevalence of oral health problems were not reported, impeding interpretation and comparison of results.

### Intra-caregiver agreement

To the best of our knowledge, intra-caregiver reliability has not been assessed previously for the oral health–related section of the MDS/interRAI. For most items, kappa values were ≥ 0.6 or only slightly lower, which represents adequate agreement [34]. The lowest kappa was found for gums (*κ* = 0.45; 82.8%). This indicates substantial problems with this item which also achieved very low dentist-caregiver- and inter-caregiver agreement.

### Effect of the video training

Caregivers who watched the video training had a higher increase in oral health–related knowledge, but only isolated and small positive effects on psychometric properties were found. Agreement with the dentist on acceptable conditions of *gums* and *tongue* was even lower for caregivers who watched the video training. In contrast, Arvidson-Bufano et al. (1996) reported higher accuracy of nurses using the MDS after a 30-minute training with hands-on practice [23]. Hoben et al. (2019) confirmed that research assistants who had attended a half-day training session identified more oral health problems and achieved a higher agreement with a dental hygienist than untrained care staff [47].

An in-person training allows to ask questions, provide feedback, and practice hands-on. However, for feasibility reasons, we opted to develop a video training. Videos can be provided online and caregivers can individually choose place, time, speed, and frequency of watching. Studies that compared in-person- to video lectures in the medical field have shown that both can be equally effective in transferring knowledge to students [49]. However, it was found that students who attended a lecture in person performed somewhat better with regard to clinical practical skills [50].

The limited differences found in our study might result from the fact that all caregivers attended a 1-hour session on the optimized ohr-interRAI section and the study procedure. It can be expected that compared to a completely uninformed group, the video training may cause larger differences. Furthermore, caregivers watched the videos only once without the option to repeat sequences.

### Interpretation of the results and future prospects

Compared to previous versions, psychometric properties of the ohr-interRAI were improved, but substantial difficulties to detect oral care needs still remain. Comments from participating dentists and caregivers indicated that the items need further refinement. However, it can be supposed that the main problem is related to a lack of training and experience of the caregivers. The differences between caregivers and dentists were substantial, even when the latter did not use any diagnostic instruments. This indicates that further training can help to raise caregivers’ abilities to detect oral care needs. The video training developed in this study might be more effective if caregivers are allowed to watch the videos on their own pace. It also needs to be considered to provide hands-on training with feedback from oral health professionals which was shown to enhance the accuracy of oral health assessments [23]. To ensure a long-lasting effect, these training sessions need to be repeated regularly [51]. However, the interRAI assessment is very comprehensive and intricate training for each individual section is not feasible. An approach to solve this problem could be intense training of a few caregivers who complete the ohr-interRAI section for the clients of several care institutions. It was shown that coaching programs with practical support from oral health professionals for individual nursing home personnel are feasible [52]. A pilot study found that after 12 hours of training, nurses were able to formulate oral care plans that were highly congruent with those of an oral health professional [53]. In addition to specific ohr-interRAI training programs, the attention for oral health in the training of healthcare students needs to be increased as well. Current curricula of nursing students often cover the topic insufficiently, but it was repeatedly shown that particularly inter-professional education is suited to improve oral health–related knowledge, competence, and confidence among nursing students [54–57].

Further refinement of the optimized ohr-interRAI should include the general condition of the client. Particularly for persons in a palliative state, the main aim of the assessment shifts towards oral comfort and analgesia, while other aspects such as painless cavities become less relevant. InterRAI registers whether a person is in a terminal state of life. In the further refined version of the optimized ohr-interRAI, this information is included in the algorithm that determines the need of necessary care interventions.

## Conclusion

An optimized ohr-interRAI section was developed with test content pre-determined by a group of experts, resulting in recommended interventions derived from the assessment. Although psychometric properties were improved compared to previous versions, the optimized ohr-interRAI section and the video training need further refinement. Subsequently, the optimized ohr-interRAI section needs to be tested within the complete interRAI assessment in an everyday care context. The current research confirmed a high prevalence of oral health problems in care-dependent older adults, stressing that alongside the refinement of the optimized ohr-interRAI section, access to regular professional dental services should be pursued as much as possible.

## Supplementary Information

ESM 1 (DOCX 7 kb)

ESM 2 (PDF 1823 kb)

ESM 3 (SAV 39 kb)

ESM 4 (MP4 66667 kb)

ESM 5 (MP4 60227 kb)

ESM 6 (MP4 69232 kb)

ESM 7 (MP4 63449 kb)

ESM 8 (MP4 83530 kb)

ESM 9 (MP4 91957 kb)

ESM 10 (MP4 69641 kb)

ESM 11 (MP4 45509 kb)

ESM 12 (MP4 53649 kb)
