# Correction to: Assessment of oral health in older adults by non-dental professional caregivers—development and validation of a photograph-supported oral health–related section for the interRAI suite of instruments

**DOI:** 10.1007/s00784-020-03725-3

**Published:** 2021-06-10

**Authors:** Stefanie Krausch-Hofmann, Trung Dung Tran, Barbara Janssens, Dominique Declerck, Emmanuel Lesaffre, Johanna de Almeida Mello, Anja Declercq, Jan De Lepeleire, Joke Duyck

**Affiliations:** 1 KU Leuven Department of Oral Health Sciences, Population Studies in Oral Health, Kapucijnenvoer 7/a - box 7001, 3000 Leuven, Belgium; 2 KU Leuven Department of Public Health and Primary Care, Biostatistics and Statistical Bioinformatics Centre (L-BioStat), Kapucijnenvoer 35/a - box 7001, 3000 Leuven, Belgium; 3 GhentUniversityDepartment of OralHealth Sciences, SpecialNeeds in Oral Health, Gerodontology, C.-Heymanslaan 10, entrance 25, 9000 Ghent, Belgium; 4 KU Leuven LUCAS, Centre for Care Research and Consultancy, Minderbroedersstraat 8 - box 5310, 3000 Leuven, Belgium; 5 KU Leuven CESO, Center for Sociological Research, Parkstraat 45 - box 3601, 3000 Leuven, Belgium; 6 KU Leuven Department of Public Health and Primary Care, Academic Centre for General Practice, Kapucijnenvoer 33/j - box 7001, 3000 Leuven, Belgium; 7 KU Leuven Department of Oral Health Sciences, Biomaterials/BIOMAT, Kapucijnenvoer 7/a - box 7001, 3000 Leuven, Belgium

**Correction to: Clinical Oral Investigations.**

10.1007/s00784-020-03669-8

After publication of this paper, the authors determined a step sequencing error under “Materials and methods”. Instead of “step 2” and “step 4” the article reads “1”. Under “Compliance with ethical standards” formatting of the sub-heading “Ethical approval” was in need of correction.

The original article has been corrected.

