# Supplementary material for: Assessment of oral health in older adults by non-dental professional caregivers—development and validation of a photograph-supported oral health–related section for the interRAI suite of instruments
Source: Clin Oral Investig. 2020 Nov 16;25(6):3475–86. doi: 10.1007/s00784-020-03669-8 (PMC8137625; doi:10.1007/s00784-020-03669-8)
Supplement: Supplementary file 1 — (DOCX 7 kb) [file 784_2020_3669_MOESM1_ESM.docx]

1. Oral health-related section that is included in the MDS 2.0 and in the interRAI versions for long-term care.

|  | MDS 2.0 | InterRAI |
| --- | --- | --- |
| Countries where the instruments are used or tested (status 03/2019) | | |
|  | North America (Canada),  Europe (Finland) | North America (Canada),  Europe (Austria, Belgium, Czech Republic, Denmark, Estonia, France, Germany, Iceland, Italy, Lithuania, Netherlands, Norway, Poland, Spain, Sweden, Switzerland, United Kingdom),  Asia and Pacific Rim (Australia, China, Hong Kong, Japan, New Zealand, Singapore, South Korea) |
| Items | | |
| Chewing | Chewing problems | Reports difficulty with chewing |
| Swallowing | Swallowing problems | Detailed evaluation in section K3 |
| Pain | Mouth pain | Reports mouth or facial pain/discomfort |
| Status oral hygiene | Debris (easily removable substances) in mouth at bedtime |  |
| Performance daily oral hygiene | Daily oral health care by resident or staff |  |
| Removable dental prosthesis use | Has dentures/removable bridge | Wears a denture/removable prosthesis |
| Tooth loss and absence removable dental prosthesis | Some/all natural teeth lost, no dentures/partial plates available/used |  |
| Condition teeth | Broken, loose, or carious teeth | Has broken, fragmented, loose, or otherwise non-intact natural teeth |
| Condition gums | Inflamed, swollen or bleeding gums, oral abscesses, ulcers, or rashes | Presents with gum (soft tissue) inflammation or bleeding adjacent to natural teeth or tooth fragments |
| Dry mouth |  | Reports having dry mouth |
