# Supplementary material for: Assessment of oral health in older adults by non-dental professional caregivers—development and validation of a photograph-supported oral health–related section for the interRAI suite of instruments
Source: Clin Oral Investig. 2020 Nov 16;25(6):3475–86. doi: 10.1007/s00784-020-03669-8 (PMC8137625; doi:10.1007/s00784-020-03669-8)
Supplement: Supplementary file 2 — (PDF 1823 kb) [file 784_2020_3669_MOESM2_ESM.pdf]

Optimized photograph-supported ohr-interRAI section

1. General utilization guidelines

Relevance

- Good oral health contributes to general health and well-being.

Aim

- Detect clients who need assistance with daily oral hygiene and/or referral to a dentist

Communication

- Inform clients that you will ask questions about the mouth and that you will look into the mouth as well.
- Talk to clients themselves. Turn to family or caregivers only if clients are not able to communicate.

Inspection of the mouth

- Ask clients to take out dentures. Help, if necessary.
- Make sure that the head of clients is supported during the inspection.
- Wear examination gloves and use flashlights for illumination.
- Ask clients to open the mouth. For better view, pull cheeks and lips away with your finger or with the handle of a toothbrush.

If you are not certain, register presence of oral health problems.

2. Definitions and guidelines per item

2.a Chewing problems

How well could you chew in the last 3 days?

- I could chew all kinds of food.
- I only had problems with hard or chewy food (e.g. nuts, raw apples, steak).
- I also had problems with soft food (e.g. cooked potatoes, banana, cake).
- Cannot be assessed/mixed food due to swallowing issues

Guidelines and definitions

- If clients don’t have or don’t wear dentures during meals, ask how chewing goes without dentures. If clients wear dentures during meals, assess chewing with the dentures.
- If food is blended/pureed due to chewing problems, register accordingly. If food is blended/pureed due to other reasons such as dysphagia, register that chewing cannot be assessed.
- If clients are not able to communicate, turn to primary caregivers and family or observe clients during meals and look out for nonverbal signs.

2.b Discomfort or pain

How often did you had discomfort or pain in the last 3 days?

- Not in last 3 days
- Not every day
- Every day
- Cannot be assessed

Guidelines and definitions

- Register discomfort or pain regardless of the underlying cause and whether appearing in rest, during meals or during oral care. Dentures might also cause discomfort or pain.
- If clients are not able to communicate, turn to primary caregivers and family or observe clients and look out for nonverbal signs.

2.c Dry mouth

How often did you had discomfort or pain in the last 3 days?

- Not in last 3 days
- Not every day
- Every day
- Cannot be assessed

Guidelines and definitions

- The mouth can feel dry in rest or during meals. Clients might also mention dry lips.
- If clients are not able to communicate, turn to primary caregivers and family or observe and look out for nonverbal signs.

2.d Denture hygiene

- <1/3 of the inner surface is covered by plaque or tartar

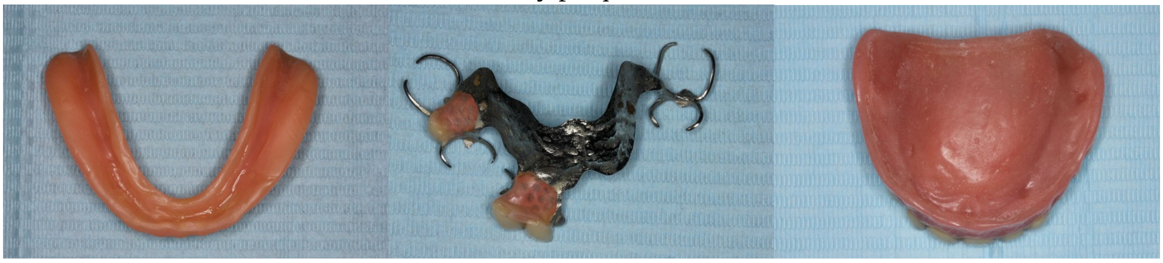

- ≥1/3 of the inner surface is covered by plaque or tartar

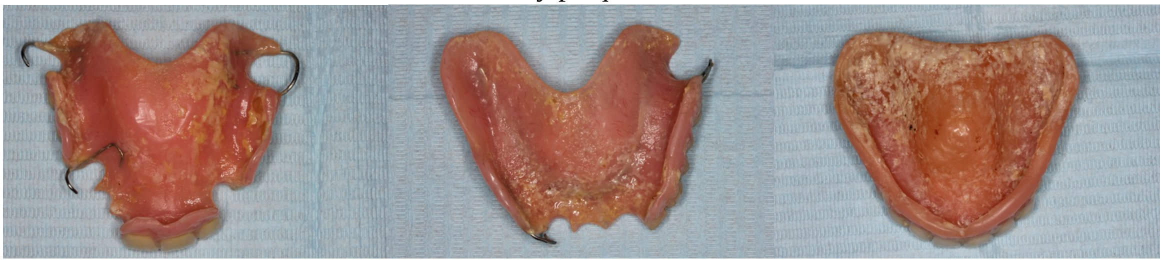

- Cannot be assessed/does not have or does not wear dentures

Guidelines and definitions

- Inner surface: surface that covers and rests on the gums.
- Plaque: sticky deposit that is white or pale yellow, can be removed with toothbrushing.
- Tartar: hard deposit that is yellow or brown, cannot be removed with toothbrushing.
- Take out dentures and rinse under water to remove food remnants.
- If clients wear dentures in upper and lower jaw, assess the denture with the poorest hygiene.

2.e Oral hygiene

- <1/3 of the surface of teeth or denture retainers is covered by plaque or tartar

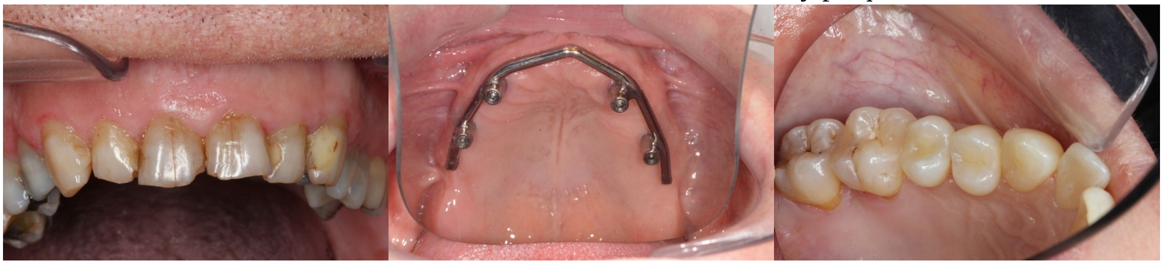

- ≥1/3 of the surface of teeth or denture retainers is covered by plaque or tartar

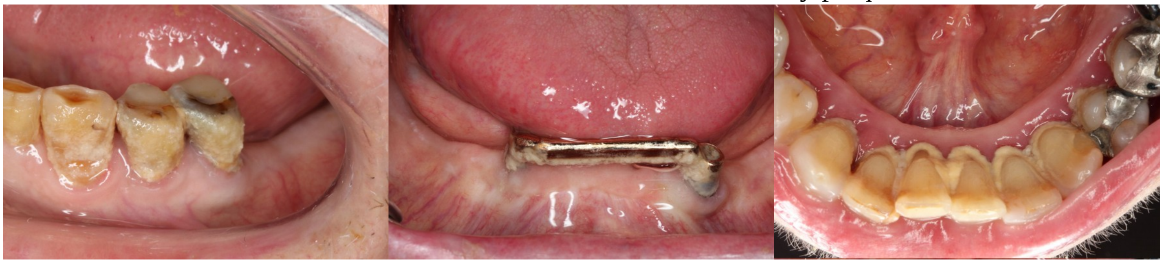

- Cannot be assessed/does not have teeth or denture retainers

Guidelines and definitions

- Denture retainers: attachments that are fixed in the mouth to anchor the denture.
- Inspect all surfaces of teeth, spaces between teeth and denture retainers.
- Assess the area of the mouth with the poorest hygiene.

2.f Teeth

- All teeth sound, adequately filled, maybe with tooth wear

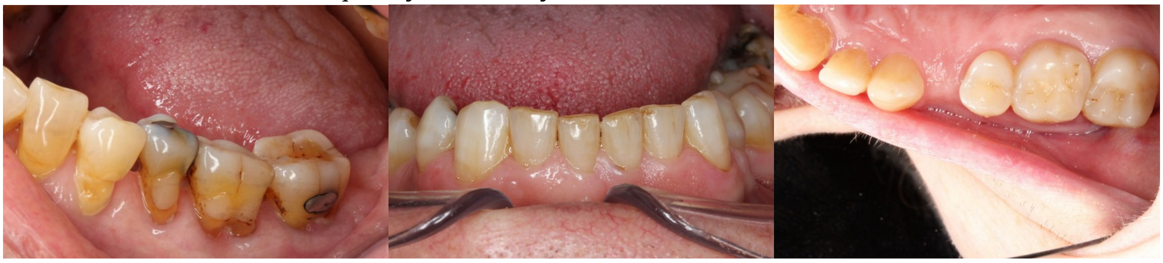

- One or more teeth broken, decayed or with defect fillings; root remnants

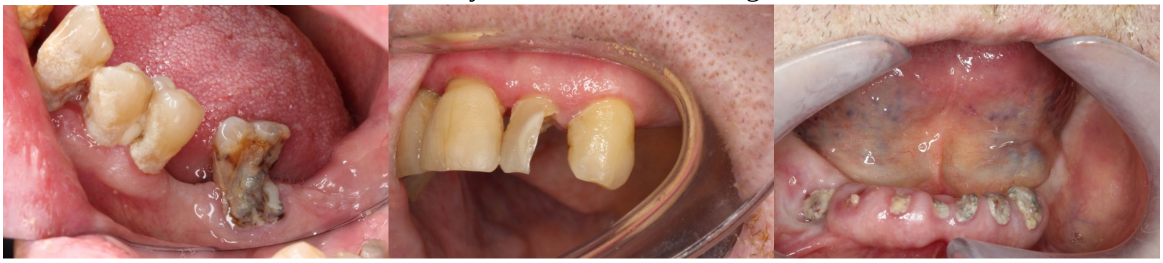

- Cannot be assessed/does not have teeth

Guidelines and definitions

- Wear: teeth evenly flat and shortened.
- Tooth decay: cavities that are stained orange or brown; large cavities can cause breakage of teeth.
- Defect filling: filling fell out or is broken; space or tooth decay at interface between tooth and filling.
- Root remnant: crown of tooth is missing; upper part of the remaining root is visible.
- Inspect the different surfaces of all teeth.

2.g Gums

- Pink and firm, maybe minor aberration in color or texture

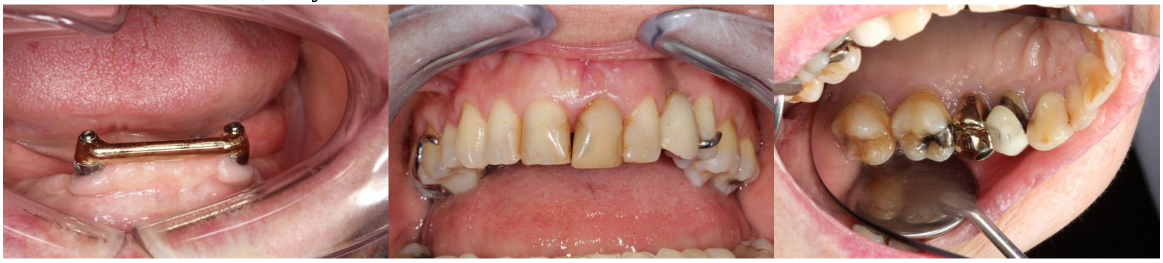

- One or more sites with redness, swelling, glassy, with sores or spontaneous bleeding

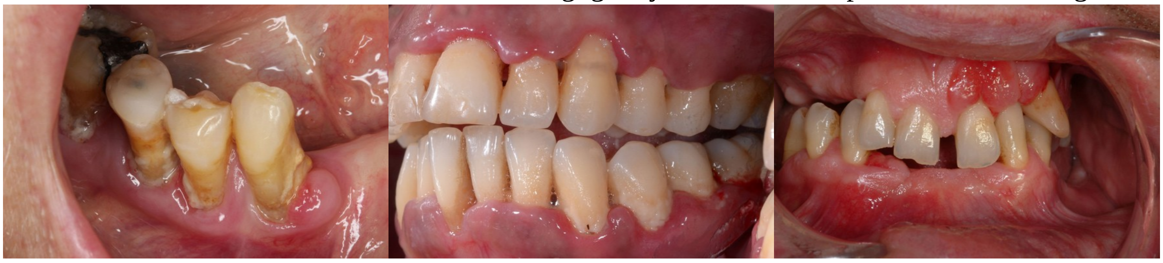

- Cannot be assessed

Guidelines and definitions

- Gums: pink tissue surrounding teeth or denture retainers.
- If clients don't have teeth or denture retainers, inspect the areas of the jaws where usually the teeth are located.
- Look out for general and localized gum problems.

2.h Tongue

- Pink, moist, healthy

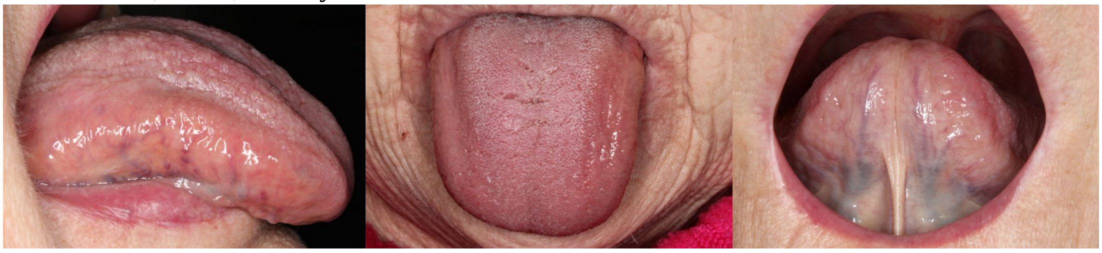

- One or more sites red, dry, swollen, with sores or patches

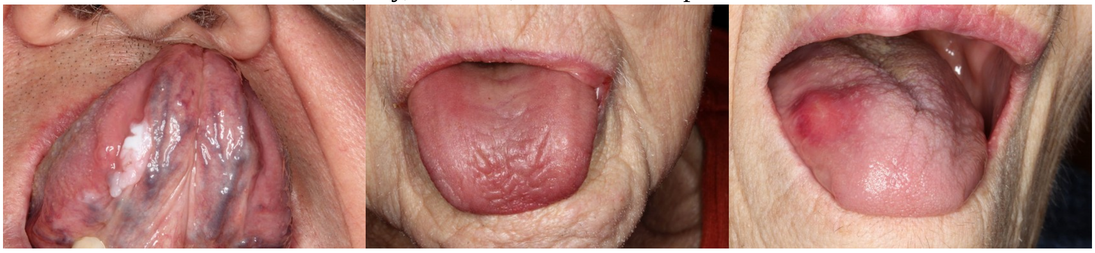

- Cannot be assessed

Guidelines and definitions

- Ask the client to extend the tongue out of the mouth. Inspect the upper and the lateral surfaces of the tongue.
- Than ask the client to curl the tongue upward to inspect the lower surface and the area under the tongue.

2.i Palate and inner surfaces of cheeks and lips

- Smooth, moist, pink

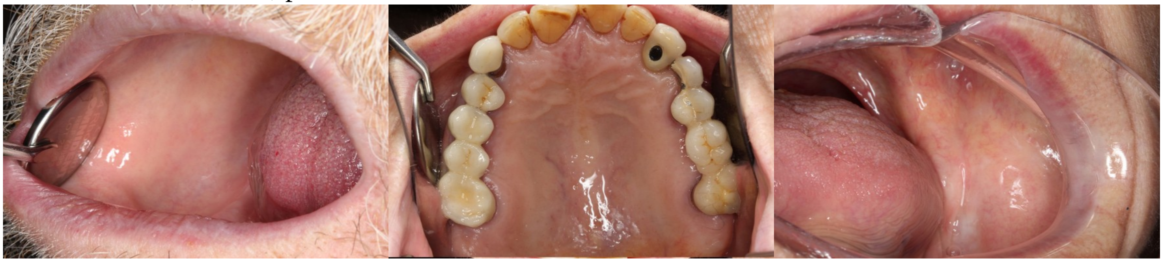

- One or more sites red, dry, swollen, with sores or patches

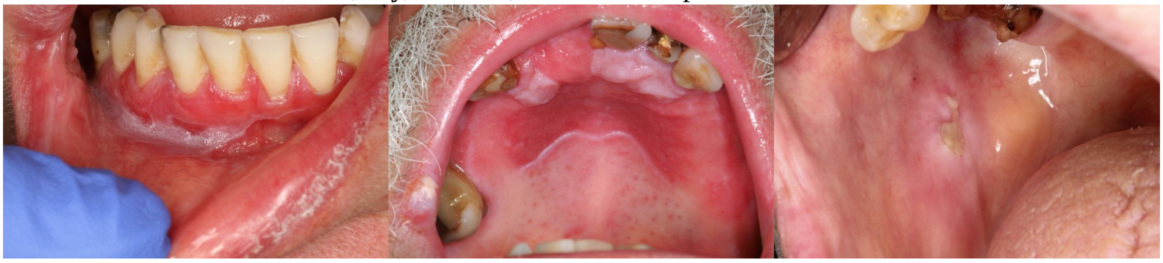

- Cannot be assessed

Guidelines and definitions

- Inspect the palate and the inner surfaces of cheeks and lips.
